# Efficacy of sensory-based static balance training on the balance ability, aging attitude, and perceived stress of older adults in the community: a randomized controlled trial

**DOI:** 10.1186/s12877-023-04596-5

**Published:** 2024-01-11

**Authors:** Yingyuan Ni, Shasha Li, Xiaoying Lv, Yuecong Wang, Lijun Xu, Yingxue Xi, Yanhua Sun, Jianyi Bao, Shufang Liao, Yue Li

**Affiliations:** 1 Department of Nursing, College of Medical Science, Huzhou University, No. 759 Second Ring Road, Huzhou City, Zhejiang Province 313000 China; 2 Department of Nursing, Huzhou Central Hospital, No. 1558 Sanhuan North Road, Wuxing District, Huzhou, Zhejiang 313000 China; 3 Department of Orthopedics, Huzhou Central Hospital, Huzhou, No. 1558 Sanhuan North Road, Wuxing District, Zhejiang 313000 China

**Keywords:** Old adult, Sensory-based static balance training, Balance ability, Aging attitude, Perceived stress

## Abstract

**Background:**

This study explores the effect of sensory-based static balance training on the balance ability, aging attitude, and perceived stress of older adults in the community. It provides a practical basis for the in-depth implementation and revision of the community health management model.

**Methods:**

A randomized controlled intervention study was conducted from 2022 to 2023. A total of 72 older adults were recruited and randomly divided into an intervention group (36 individuals) and a control group (36 individuals). Balance ability (measured by the Short Physical Performance Battery and One Leg Stand Test), aging attitudes, and perceived stress were assessed at baseline and at the 12-week and 24-week follow-ups. Repeated-measures ANOVA and generalized estimating equations were used to compare outcome measures.

**Results:**

Sensory-based static balance training was beneficial for balance ability and aging attitude among participants in the intervention group. At the end of the intervention, participants in the intervention group showed significant improvements in SPPB scores and OLST scores compared with the control group (*F*_SPPB_ = 12.347, *P* = 0.001, Wald*χ*^2^_OLST_ = 45.530, *P* < 0.001), as well as significant differences in aging attitudes (*F*_AAQ_ = 18.549, *P* < 0.001). Multiple comparisons at different time points in the intervention group reveal a significant intervention effect (*F*_SPPB_ = 29.211, Wald*χ*^2^_OLST_ = 80.428, *F*_AAQ_ = 45.981, all *P* < 0.05). However, the difference in perceived stress before and after the intervention was not significant (*F*_CPSS_ = 2.876, *P* = 0.095).

**Conclusions:**

Sensory-based static balance training significantly improved balance ability and aging attitudes among older adults in the community. The effect on perceived stress among older adults in the community was not significant.

**Trial registration:**

Registered in the Chinese Clinic on 04/06/2022. The registration number is ChiCTR2200060541.

## Introduction

Delaying functional decline and promoting mental health have become important for health promotion programs among older adults [1, 2]. Balance ability is an indicator of older adults' functional decline and is essential for their independent completion of activities of daily living (ADLs) [3]. Studies have demonstrated that the balance ability of older adults progressively declines with age [4]. Up to the age of 60, balance ability can remain relatively intact; however, it gradually diminishes at an approximate rate of 16% every decade thereafter [5]. The decline in balance significantly increases the risk of falls among older adults and impairs their capacity to effectively perform ADLs [6–8], thus increasing their negative aging attitudes and perceived stress [9, 10].

Aging attitudes encompass individuals' subjective experiences and evaluations of the aging process and old age, representing a multifaceted psychological state [11]. The prevailing level of aging attitude among older adults in the community is moderately satisfactory but necessitates further enhancement [12]. Positive attitudes significantly contribute to stabilizing older adults' emotional well-being and promoting their engagement in active physical exercise [13, 14]. Conversely, negative attitudes are closely associated with adverse psychological states experienced by older adults [15], such that more negative perceptions of aging correspond to heightened levels of perceived stress [16].

Furthermore, perceived stress denotes a psychological imbalance arising from cognitive appraisal of an individual's response to stressful events, primarily manifesting as tension or loss thereof [17, 18]. Older adults' perceived stress is intricately linked with their perceived functional health status and aging attitude [19, 20]. Changes in perceived stress are influenced by the functional health status of older adults [21, 22]. Consequently, prioritizing balance ability within older adults in the community is of paramount importance for fostering psychosocial well-being.

Task-specific balance training is considered the optimal intervention for enhancing balance in older adults [23]. Balance training encompasses static or dynamic exercises conducted on stable or unstable surfaces, as well as active and reactive training to improve balance control [24]. Notably, balance training significantly enhances objective monitoring indicators among older adults, including improved scores on the Short Physical Performance Battery (SPPB), Timed Up-and-Go Test (TUG), Berg Balance Scale (BBS), One Leg Stand Test (OLST), and Functional Reach Test (FR) [23, 25, 26]. Static balance training can effectively enhance the balance and functional ability of stroke patients. However, the application of static balance training in older adults in the community has not been promoted to date [27, 28]. Recent research findings have highlighted vision and hearing as influential factors affecting the balance of older adults [29]. Consequently, there remains a need to explore the efficacy of static balance training based on sensory changes specifically within older adults in the community.

Overall, this study is rooted in social-ecological theory and incorporates elements such as functional exercise characteristics, learning ability, and community environment specific to older adults. It aims to develop a sensory-based static balance training program targeting older adults in the community. The objectives of our study aim to examine the effects of this program on balance ability, aging attitudes, and perceived stress among older adults in the community. This research provides both theoretical and practical insights for developing active aging health promotion programs tailored to senior adults residing in communities.

Based on previous studies, the hypotheses of the study were as follows. Hypothesis 1: Sensory-based static balance training will improve balance ability among older adults in the community, as measured by the Short Physical Performance Battery (SPPB) and One Leg Stand Test (OLST). Hypothesis 2: Sensory-based static balance training will improve aging attitudes and perceived stress.

## Materials and methods

### Study design

This study used an assessor-blinded, randomized, controlled, parallel arm test design. Approval for the protocol was obtained from the Medical Ethics Committee of Huzhou University (NO.2022–09-22). The study was conducted in accordance with the ethical principles of the Declaration of Helsinki. Each participant was informed of the aims and details of the research, and written consent was obtained from all old adults.

### Participants

The trial was conducted from October 2022 to March 2023 in the northern part of Zhejiang Province, China. The inclusion criteria were as follows: (i) older adults living in the community for more than one year; (ii) age greater than or equal to 60 years old; (iii) able to live and walk independently, with an SPPB score greater than or equal to 7; (iv) no communication barriers; and (v) informed consent to participate in the intervention and willingness to cooperate. The exclusion criteria were as follows: (i) older adults who were not permanent residents of the community; (ii) had participated in balance training programs; (iii) suffered from chronic diseases that were not stable; and (iv) took medications that could affect balance and mental status.

### Sample size

The sample size was analyzed using G*Power version 3.1 for power analysis. The t test set 1-β at 0.8 and significance level at 0.05. Based on similar studies and relevant data from Jacobson [30], an effect size (d) of 0.761 and a total sample size of 58 were established. Considering a potential attrition rate of 25%, this study recruited 72 older adults, including 36 each in the intervention group and the control group.

### Randomization and blinding

To avoid potential contamination during the intervention, older adults were selected from two geographically distant communities. The research assistant was unfamiliar with the intervention plan and was only responsible for participant eligibility assessment and data collection. An independent researcher will write slips of paper labeled "Intervention Group" and "Control Group" and place them into envelopes. Representatives from both communities randomly draw envelopes. The community that draws the envelope labeled "Intervention Group" will be assigned as the intervention group, while the remaining community will serve as the control group. Recruited participants and the intervention facilitator were blinded to the group assignment.

### Intervention

#### Intervention plan

**Fig. 1:**
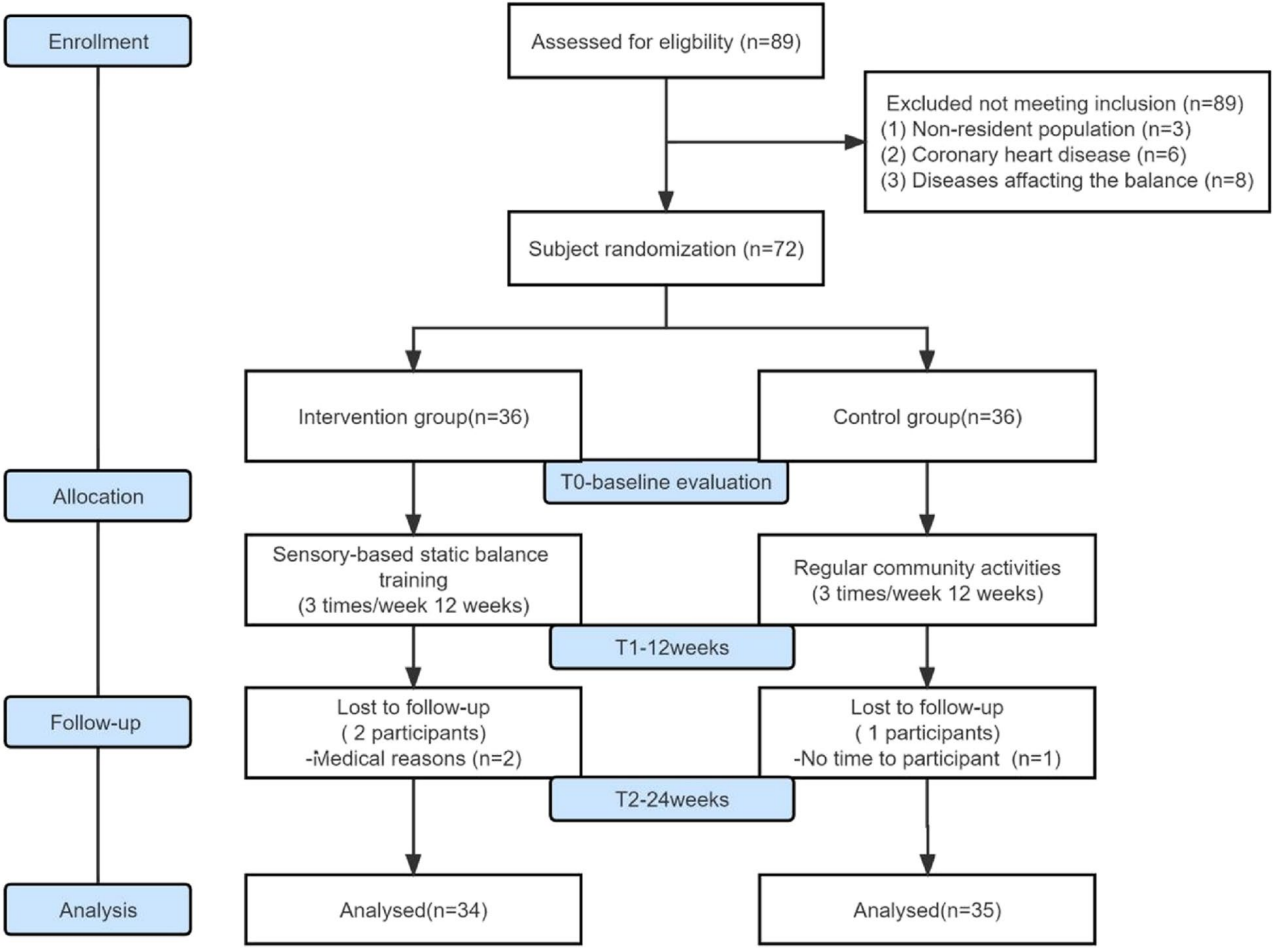
Study flow chart

Thirty-six older adults in the intervention group were randomly divided into three groups of 12 by drawing lots with 1 coach and 2 supervisors. The appropriate time (9:00–10:00 am) was selected according to the activity habits of older adults in the community. Each group participated 3 times a week for 40 min each time for 12 weeks, and a follow-up was completed at 24 weeks. See Fig. 1 for details.

The training program is based on the characteristics of acquisition and reinforcement, simple to complex, using a one-week teaching and one-week training model to increase the difficulty of training by reducing sensory input and increasing external interference. The intervention program was reviewed by five experts in rehabilitation kinesiology, medical psychology, geriatrics, health management, and nursing psychology. They provided feedback on the recommendations, and the program was revised accordingly. Sensory-based static balance training action was combined with multicomponent media changes, such as contact surface, audiovisual and external interference, to form a sensory-based static balance training program. See Table 1 for details.

**Table 1:**
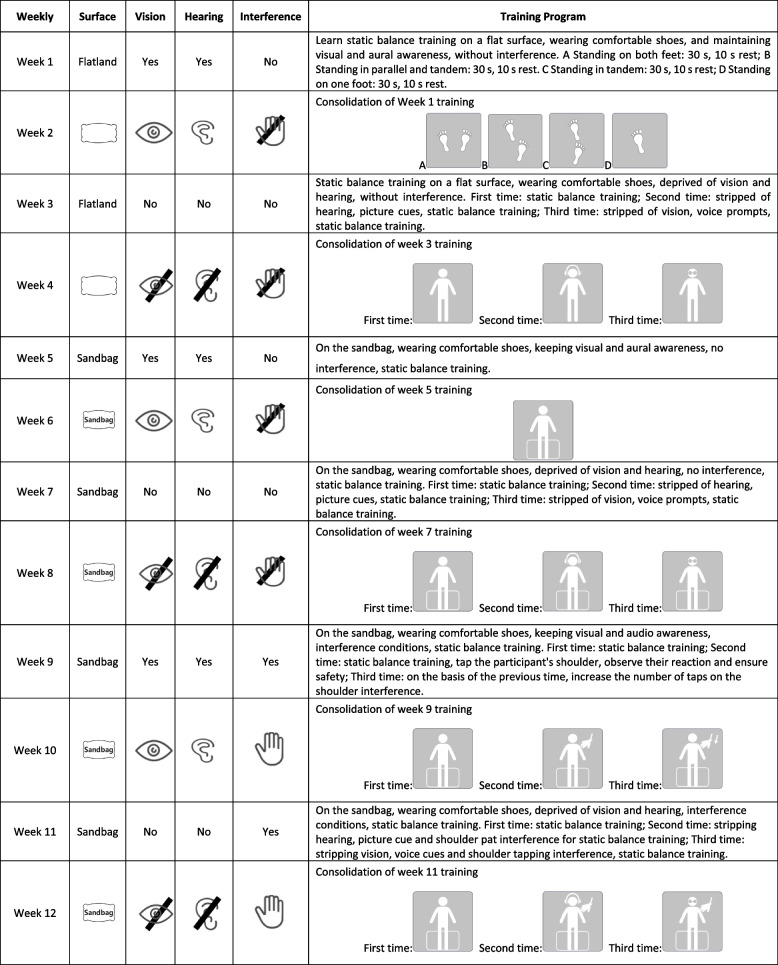
Static balance training program

**Fig. 2:**
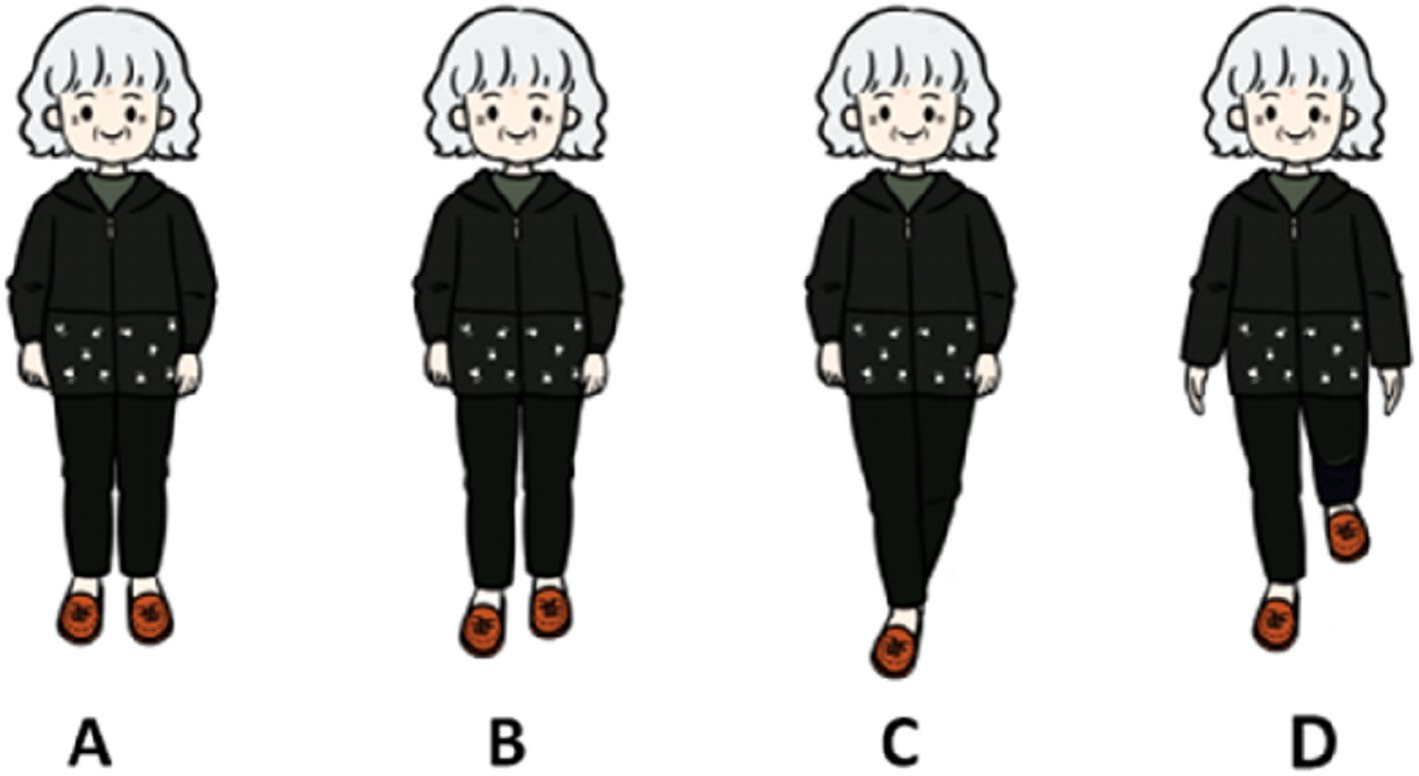
Exercise movements. Note: (**A**) standing on both feet; (**B**) standing on both feet semi-back and forth; (**C**) standing on both feet in a straight line back and forth; (**D**) standing on one foot

During sensory-based static balance training, the posture of older adults when stationary is detailed in Fig. 2. (A) Standing on both feet; (B) standing on both feet semiback and forth; (C) standing on both feet in a straight line heel to toe; (D) standing on one foot. The intervention program consisted of 6 groups of training programs: (i) basic static balance training; (ii) static balance training on a flat surface under conditions of audio-visual deprivation and no interference; (iii) static balance training on a sandbag under conditions of audio-visual preservation and no interference; (iv) static balance training on a sandbag under conditions of audio-visual deprivation and no interference; (v) static balance training on a sandbag under conditions of audio-visual preservation and interference; and (vi) static balance training on a sandbag under conditions of audio-visual deprivation and interference. The specific program is shown in Table 1.

#### Intervention quality control

The interventionists underwent standardized training, and the intervention team was composed of three coaches trained in sensory-based static balance training and six graduate nursing students. A team of experts composed of a clinical rehabilitation medicine specialist, a senior rehabilitation technician, and a community geriatrician was invited to design and oversee the overall intervention as a field team.

#### Control group

The control group had regular exercise-related health education one time per week for 40 min each. The intervention lasted for 12 weeks, and a follow-up visit was scheduled.

### Assessment

#### Sociodemographic profile

The sociodemographic profile included age, sex, education level, marital status, living condition, weekly exercise frequency, self-assessed health and mood.

#### Short Physical Performance Battery (SPPB)

The SPPB consists of three domains to measure balance, mobility, and lower body muscle strength: the balance test (BT), gait speed (GS) test, and the chair sit-to-stand (STS) test, respectively. Part 1: This part of the test requires the participant to perform a "semi-tandem" movement, and if the participant can hold the position for 10 s, to perform a "tandem stand" movement. Otherwise, the participant is asked to perform a "stand with feet together" movement. Part 2: This part of the test requires the participant to walk 3 m in a defined area twice. The faster results is used for data analysis. Part 3: This part of the test requires the participants to fold their arms across their chest and perform five chair stands as quickly as possible. [31]. Higher SPPB scores indicate better physical function. The test is primarily used to assess functional capacity (especially to assess lower extremity mobility) in older adults in hospitals and in the community [32].

#### One Leg Stand Test (OLST)

The OLST required the participant to raise one leg (either is allowed), stand on the remaining leg, keep his or her eyes open and look forward for as long as possible. As soon as the participant lifted his leg, the timing began. When the participant dropped the lifted foot to the ground, the timing stopped. In general, 60 s or more is regarded as good, 30 s to 60 s is typical, and 30 s or less is bad. The shorter the duration is, the weaker the participant's ability to balance [33].

#### Attitudes to Aging Questionnaire (AAQ)

The Attitudes to Aging Questionnaire (AAQ) was developed by Laidlaw et al. and was translated and adapted into the Chinese version of the Aging Attitudes Questionnaire by Huang Yifan et al. [34]. The AAQ consists of three dimensions: psychosocial loss, physiological change, and psychological growth, each with 8 items, and all items were scored on a 5-point Likert scale (ranging from 1 "not at all true" to 5 "completely true"). Among them, psychosocial loss is a negative dimension, with higher scores indicating more negative aging attitudes. In this study, the negative dimension used reverse scoring. The higher the AAQ score is, the more positive the aging attitude. The Cronbach's alpha coefficient was 0.82 [35].

#### Chinese Perceived Stress Scale (CPSS)

The Chinese Perceived Stress Scale (CPSS) was revised by Tingzhong Yang et al. and contains 2 dimensions, tension and loss of control, and all items were rated on a 5-point Likert scale (from 1 to 5 for "never" to "always", respectively). Higher scores indicated more perceived stress. The total score ranges from 14 to 70. The Cronbach's alpha coefficient was 0.88 [36].

### Statistical analysis

SPSS 25.0 statistical software was applied for data entry and statistical analysis, the statistical inference alpha value was taken at a test level of 0.05, and two-sided *P* values were calculated. Before data analysis, the coding and entry of data were checked for errors, omissions, and logic. Categorical and continuous variables were compared using chi-square analysis and independent samples *t* tests, respectively. This study used a two-factor repeated-measure experimental design with three variables. The between-group variable was the intervention group versus the control group. The within-group variable was the number of tests, and the test was repeated three times in the study at preintervention T0, postintervention T1, and the 3-month follow-intervention T_2_. The dependent variables were SPPB, OLST, AAQ, and CPSS scores, all of which were continuous variables. The distributions of SPPB, AAQ and CPSS scores were tested for normality. A repeated-measures ANOVA was used to compare the changes in SPPB, AAQ, and CPSS scores before and after the intervention in both groups after controlling for baseline values of specific outcomes. Because the BT, GS, STS and OLST scores did not show normal and approximately normal distributions, we used generalized estimating equations (GEEs) for the analysis. If significant interaction effects were observed, further analysis should be conducted using simple effect and Bonferroni post hoc test analysis to examine the time and group factors. This is done to determine the specific time point at which the intervention produces effects.

## Results

### Baseline characteristics

The baseline characteristics of the study participants are shown in Table 2. The majority of the older adults in this study were 60–70 years old (59.9%, 39/69). There were no significant differences in baseline sociodemographic characteristics between the intervention and control groups (*P* > 0.05).

**Table 2:** Sociodemographic Characteristics of the Participants in the Two Groups

Sociodemographic Characteristics	Intervention Group (*n* = 34)	Control Group (*n* = 35)	*X*^2^ value	*P* value
Age, n (%)			0.476	0.826
60–70	18(52.9)	21(60.0)		
71–80	12(35.3)	11(31.4)		
> 80	4(11.8)	3(8.6)		
Sex, n (%)			0.026	0.873
Male	12(35.3)	13(37.1)		
Female	22(64.7)	22(62.9)		
Education level, n (%)			2.649	0.266
Primary school and less	19(55.9)	13(37.1)		
Junior school	7(20.6)	12(34.3)		
High school and more	8(23.5)	10(28.6)		
Marital status, n (%)			0.749	0.387
Married	29(85.3)	27(77.1)		
Unmarried and Married (divorced or widowed)	5(14.7)	8(22.9)		
Living conditions, n (%)			0.749	0.387
Nonsolitary	29(85.3)	27(77.1)		
Solitary	5(14.7)	8(22.9)		
Weekly exercise frequency, n (%)			1.489	0.475
< 3 times/week	15(44.1)	15(42.9)		
3–4 times/week	5(14.7)	9(25.7)		
> 4 times/week	14(41.2)	11(31.4)		
Health self-assessment, n (%)			0.189	0.797
Normal	10(29.4)	12(34.3)		
Good	29(85.3)	23(65.7)		
Mood self-assessment, n (%)			2.708	0.153
Normal	5(14.7)	11(31.4)		
Good	29(85.3)	24(68.6)		

### Intervention effects

All outcome indicators had group and time interactions except GS (Wald χ2 = 5.784, *P* = 0.055) and CPSS (F = 1.103, *P* = 0.338) scores (see Table 3). These results indicated that sensory-based static balance training was not effective in improving gait speed and perceived stress in older adults in the community.

**Table 3:** Impact of the Intervention on Outcome Measures at Three Timepoints (Group × Time) test (*N* = 69)

Outcome Measures	Measure Time			Group Factor Effect	Time Factor Effect	Group × Time Effect
	T_0_ (Pretest)	T_1_ (Posttest)	T_2_ (Follow-test)	*F*/Wald *χ*^*2*^	*F*/Wald *χ*^*2*^	*F*/Wald *χ*^*2*^
	Mean T SD	Mean T SD	Mean T SD	*P* -value	*P* -value	*P*- value
**SPPB**				4.402 0.040	14.109 0.000	15.547 0.000
Intervention group (G_1_)	8.82 ± 1.55	9.94 ± 1.28	9.71 ± 1.38			
Control group (G_2_)	8.80 ± 1.59	8.77 ± 1.48	8.77 ± 1.47			
**BT**				30.980 0.000	9.262 0.010	45.911 0.000
Intervention group (G_1_)	3.24 ± 0.12	3.97 ± 0.03	3.79 ± 0.07			
Control group (G_2_)	3.26 ± 0.12	3.00 ± 0.08	2.97 ± 0.12			
**GS**				0.596 0.440	5.741 0.057	5.784 0.055
Intervention group (G_1_)	2.68 ± 0.14	2.71 ± 0.14	2.76 ± 0.14			
Control group (G_2_)	2.69 ± 0.13	3.30 ± 0.11	2.80 ± 0.10			
**STS**				1.733 0.188	4.293 0.117	8.677 0.013
Intervention group (G_1_)	2.91 ± 0.16	3.26 ± 0.15	3.15 ± 0.15			
Control group (G_2_)	2.86 ± 0.18	2.77 ± 0.15	2.89 ± 0.15			
**OLST**				30.780 0.000	80.073 0.000	80.515 0.000
Intervention group (G_1_)	10.41 ± 9.62	31.50 ± 20.63	27.15 ± 19.40			
Control group (G_2_)	7.34 ± 5.32	7.31 ± 5.03	7.31 ± 5.02			
**AAQ**				12.042 0.001	23.296 0.000	24.114 0.000
Intervention group (G_1_)	79.32 ± 10.09	84.76 ± 7.91	84.62 ± 7.86			
Control group (G_2_)	74.71 ± 11.17	74.63 ± 11.29	74.49 ± 11.28			
**Psychosocial loss**				7.679 0.007	7.535 0.001	5.591 0.006
Intervention group (G_1_)	32.24 ± 5.96	33.91 ± 4.48	33.76 ± 4.28			
Control group (G_2_)	29.83 ± 6.13	29.74 ± 5.94	29.57 ± 5.77			
**Physical change**				3.648 0.060	10.345 0.000	6.239 0.003
Intervention group (G_1_)	24.65 ± 5.66	26.71 ± 4.78	26.68 ± 4.73			
Control group (G_2_)	23.26 ± 6.39	23.49 ± 6.40	23.43 ± 6.41			
**Psychological growth**				3.785 0.056	9.616 0.000	11.990 0.000
Intervention group (G_1_)	22.41 ± 4.13	24.15 ± 3.28	24.18 ± 3.27			
Control group (G_2_)	21.60 ± 5.43	21.51 ± 5.03	21.49 ± 4.99			
**CPSS**				2.876 0.095	1.773 0.178	1.103 0.338
Intervention group (G_1_)	33.53 ± 6.35	32.29 ± 4.98	32.41 ± 4.44			
Control group (G_2_)	34.97 ± 5.19	34.83 ± 5.61	34.80 ± 5.26			

*G* group, *G1* intervention group, *G2* control group, *SPPB* Short Physical Performance Battery, *BT* Balance Test, *GS* Gait Speed, *STS* Chair Sit-to-stand, *OLST* One Leg Stand Test, *AAQ* Attitudes to Aging Questionnaire, *CPSS* Chinese Perceived Stress Scale

As shown in Table 4, there was no significant difference between the intervention group and the control group at the T_0_ time point (all *P* > 0.05). At the T_1_ time point, all outcome indicators were significantly different between the two groups (all *P* < 0.05). At the T_3_ time point, except for STS (*P* = 0.219), the other outcome indicators still showed significant differences (*P* < 0.05).

**Table 4:** Simple effect analysis and multiple comparisons of group

Outcome Measures		T0			T1			T2		
		Difference (95% CI)	F/Waldχ2	*P* -*value*	Difference (95% CI)	F/Waldχ2	*P* -*value*	Difference (95% CI)	F/Waldχ2	*P* -*value*
SPPB	G1G2	0.02(-0.73,0.78)	0.004	0.950	1.17(0.51,1.83)	12.347	0.001*	0.93(0.25,1.62)	7.353	0.008*
BT	G1G2	-0.02(-0.36,0.31)	0.016	0.898	0.97(0.80,1.14)	127.817	0.000*	0.82(0.54,1.10)	33.348	0.000*
STS	G1G2	0.06(-0.42,0.53)	0.050	0.823	0.49(0.09,0.90)	5.704	0.017*	0.26(-0.16,0.68)	1.511	0.219
OLST	G1G2	3.07(-0.56,6.70)	2.749	0.097	24.19(17.16,31.21)	45.53	0.000*	19.83(13.20,26.46)	34.386	0.000*
AAQ	G1G2	4.61(-0.51,9.73)	3.228	0.077	10.14(5.44,14.83)	18.549	0.000*	10.13(5.47,14.80)	18.779	0.000*
Psychosocial loss	G1G2	2.41(0.50,5.31)	2.735	0.103	4.17(1.63,6.70)	10.78	0.002*	4.19(1.75,6.64)	11.699	0.001*
Physical change	G1G2	1.40(-1.51,4.30)	0.913	0.343	3.22(0.50,5.94)	5.574	0.021*	3.24(0.53,5.96)	2.389	0.020*
Psychological growth	G1G2	0.81(-1.49,3.11)	0.497	0.483	2.63(0.59,4.68)	6.603	0.012*	2.69(0.66,4.73)	6.974	0.010*

*Abbreviations*: *G* group, *G*_1_ intervention group, *G*_2_ control group, *T* time point, *T*_0_ pretest, *T*_1_ posttest 1, *T*_2_ follow-test 2, *SPPB* Short Physical Performance Battery, *BT* Balance Test, *GS* Gait Speed, *STS* Chair Sit-to-stand, *OLST* One Leg Stand Test, *AAQ* Attitudes to Aging Questionnaire, * indicates a significant difference (p ≤0 .05)

Table 5 shows a significant difference in the total SPPB, BT, STS and OLST scores of the intervention (*P*_SPPB_ < 0.001, *P*_BT_ < 0.001, *P*_STS_ = 0.002, *P*_OLST_ < 0.001), while no significant differences were observed within the control group across different time points (all *P* > 0.05). Specifically, the SPPB, BT, STS and OLST scores at T_0_ were significantly lower than those at T_1._ (*P*_SPPB_ < 0.001, *P*_BT_ < 0.001, *P*_STS_ = 0.001, *P*_OLST_ < 0.001). These findings indicate that the SPPB, BT, STS and OLST scores of older adults improved following the intervention. However, the STS scores were not significantly different between T_1_ and T_2_ (*P*_STS_ = 0.435). The SPPB, BT and OLST scores were significantly different between T_1_ and T_2_ (*P*_SPPB_ = 0.002, *P*_BT_ = 0.021, *P*_OLST_ < 0.001). These findings indicate that the long-term effect on SPPB and STS after intervention was not significant, but after the intervention, the BT and OLST had a good long-term effect.

**Table 5:** Simple effect analysis and multiple comparisons of time

Outcome Measures		Intervention Group	F/Waldχ2	*P* Value	Control Group	F/Waldχ2	*P* Value
		Difference (95% CI)			Difference (95% CI)		
SPPB	T		29.211	0.000*		0.022	0.978
	T0 T1	-1.12(-1.48, -0.76)		0.000*	0.03(-0.33,0.38)		1.000
	T0 T2	-0.88(-1.22,-0.55)		0.000*	0.03(-0.30,0.36)		1.000
	T1 T2	0.24(-0.07,0.40)		0.002*	0(-0.16,0.16)		1.000
BT	T		37.698	0.000*		11.789	0.003*
	T0 T1	-0.74(-1.02,-0.45)		0.000*	0.26(0.01,0.50)		0.035*
	T0 T2	-0.56(-0.83,0.29)		0.000*	0.29(0.04,0.53)		0.017*
	T1 T2	0.18(0.02,0.33)		0.021*	0.03(-0.28,0.34)		1.000
STS	T		12.258	0.002*		0.98	0.613
	T0 T1	-0.35(-0.59,-0.11)		0.001*	0.09(-0.18,0.35)		1.000
	T0 T2	-0.24(-0.48,0.01)		0.065	-0.03(-0.27,0.22)		1.000
	T1 T2	0.12(-0.08,0.31)		0.435	-0.11(-0.40,0.17)		1.000
OLST	T		80.428	0.000*		0.091	0.763
	T0 T1	-21.09(-26.72,-15.46)		0.000*	0.03(-0.02,0.26)		1.000
	T0 T2	-16.72(-21.71,-11.76)		0.000*	0.03(-0.02,0.26)		1.000
	T1 T2	4.35(1.70,7.00)		0.000*	0.00(0.00,0.00)		1.000
AAQ	T		45.981	0.000*		0.775	0.465
	T0 T1	-5.44(-6.85,-4.03)		0.000*	0.09(-1.30,1.50)		1.000
	T0 T2	-5.30(-6.75,-3.81)		0.000*	0.23(-1.20,1.66		1.000
	T1 T2	0.15(-0.14,0.43)		0.626	0.14(-0.14,0.42)		0.645
Psychosocial loss	T		11.514	0.000*		1.467	0.238
	T0 T1	-1.68(-2.60,0.76)		0.000*	0.09(-0.82,0,99)		1.000
	T0 T2	-1.53(-2.50,0.57)		0.001*	0.26(-0.69,1.21)		1.000
	T1 T2	0.15(-0.10,0.40)		0.450	0.17(-0.07,0.42)		0.269
Physical change	T		15.615	0.000*		0.753	0.475
	T0 T1	-2.06(-2.96,-1.16)		0.000*	-0.23(-1.12,0.66)		1.000
	T0 T2	-2.03(-2.94,-1.12)		0.000*	-0.17(-1.07,0.73)		1.000
	T1 T2	0.03(-0.11,0.16)		1.000	0.06(-0.08,0.19)		0.883
Psychological growth	T		21.164	0.000*		0.005	0.995
	T0 T1	-1.74(-2.39,-1.08)		0.000*	0.09(-0.56,0.73)		1.000
	T0 T2	-1.77(-2.44,-1.10)		0.000*	0.11(-0.55,0.78)		1.000
	T1 T2	-0.03(-0.21,0.15)		1.000	0.03(-0.15,0.20)		1.000

*Abbreviation*s: *G* group, *G*_1_ intervention group, *G*_2_ control group, *T* time point, *T*_0_ pretest, *T*_1_ posttest 1, *T*_2_ follow-test 2, *SPPB* Short Physical Performance Battery, *BT* Balance Test, *GS* Gait Speed, *STS* Chair Sit-to-stand, *OLST* One Leg Stand Test, *AAQ* Attitudes to Aging Questionnaire, *CPSS* Chinese Perceived Stress Scale, * indicates a significant difference (p ≤0 .05)

Table 5 shows that there were significant differences in the total AAQ score and dimensional scores of the intervention group at different times (all *P* < 0.05), and there were no differences in the AAQ and the scores of each dimension of AAQ in the control group at different times (all *P* > 0.05). The total AAQ scores and dimensional scores at T_0_ were significantly lower than those at T_1_ and T_2_ (all *P* < 0.05), but there was no significant difference between T_1_ and T_2_ (all *P* > 0.05). The results showed that older adults' aging attitudes improved after the intervention, and the long-term growth effect was still significant. These results indicate that the impact of intervention plans on AAQ scores is mainly achieved through influencing the three dimensions together.

A total of 72 older adults in the community were recruited for the study, each receiving the planned intervention. During the follow-up period, 2 participants in the intervention group dropped out, resulting in an effective follow-up rate of 94.4%. In the control group, 1 participant dropped out, yielding an effective follow-up rate of 97.2%, as shown in Fig. 1.

## Discussion

This study used the SPPB and OLST as important indicators for evaluating the functional status of balance ability in older adults, which expanded upon previous research findings [23, 37]. The implementation of sensory-based static balance training has been shown to yield significant improvements in balance ability (*p* < 0.01). We found that the improvement in balance ability was mainly due to the improvement in lower limb muscle strength and balance control force. The reason for this may be due to the design of sensory-based static balance training, which includes four standing postures (standing on both legs, semibilateral standing, serial standing, and one-legged standing). Training designs that progressively reduced the support base needed older adults to use more lower-limb muscles to maintain balance. Through training, the muscle strength of older adults can be improved. We used sandbags to establish a nonuniform contact surface. Training on both flat and sandy surfaces increased the difficulty coefficient, resulting in balance control force gain [38].

Noteworthy, the results of OLST and BT used to evaluate the main indicators of static standing balance showed a significant improvement in the static balance ability of older adults in the intervention group (*P* < 0.001), and showed obvious long-term sustained effect. These results may be due to the increasing difficulty factor of intermittent visual and auditory deprivation in the intervention program. Intermittent visual deprivation training aids older adults in distinguishing between somatosensory and vestibular sensory inputs, reducing overreliance on vision and improving their reactive ability [39, 40]. In addition, our study intermittently deprived older adults of auditory input during training, and older adults were more focused on muscle strength and balance training [29]. Thus, sensory-based static balance training is conducive to improving older adults's important perception of balance training more accurately.

Sensory-based static balance training can enhance the psychological growth of older adults in the community, with improvements in three aspects: psychological and social losses, physiological changes, and psychological growth. Psychological growth refers to the positive experiences of wisdom or growth that older adults acquire as they age [37]. Considering the slower learning speed and weaker ability to accept new movements in older adults, the training model of one week of learning and one week of consolidation was used in this study. Such a training mode effectively prevented negative emotions caused by difficult learning balance movements in older adults. By acquiring and practicing sensory-based static balance movements, older adults were able to gain a positive experience and knowledge of health balance functional training [41]. With the enhancement of functional status resulting from sensory-based static balance training, older adults are able to effectively engage in various activities and adeptly tackle life challenges, thereby augmenting their positive psychological experiences and growth [42].

To our astonishment, sensory-based static balance training did not have a significant impact on the perceived stress of older adults in the community (*P* > 0.05), which is not consistent with previous study findings [43]. The reason may be that the static balance training lasted only 12 weeks, and the time was too short to improve the perceived stress of older adults in the community. Studies have shown that the main sources of perceived stress for older adults in the community are major diseases, obesity, sleep disorders, and ideological conflicts [10, 44]. Sensory-based static balance training cannot fundamentally solve the above problems. Therefore, further research is needed on the impact of static balance training on perceptual stress.

Interestingly, all older adults in the intervention group completed sensory-based static balance training, with two individuals lost to follow-up due to illness. This demonstrates a high level of compliance among older adults. The reason for this may be related to the reasonable difficulty level, lifelike movements, and diversified training scenarios of sensory-based static balance training. A reasonable difficulty level is an important prerequisite for older adults to learn and accept training. Lifelike movements are beneficial for older adults to continue their self-practice in the community [45]. Diversified training scenarios can enhance older adults' training interest. In addition, the intervention training items (blindfolds, headphones) and community environments (sand and flat ground) enhance the feasibility of static balance training for older adults in the community.

## Limitations

Several limitations should be noted. First, only the OLST and balance test in SPPB were selected for the measurement of static balance ability. To increase the comprehensiveness of balance assessment in older adults, more indicators related to dynamic balance measurement should be selected to improve the measurement of balance ability in the future. Second, the sample size comes from a single city in a country. To increase the generalizability of the results, future research should employ samples from other regions. Third, although baseline data were comparable between the two study groups, confounding factors could have interfered with the study results.

## Conclusion

The study confirmed that sensory-based static balance training intervention improved the balance ability and aging attitude of older adults in the community and demonstrated the sustainability of balance ability training effects. However, the effect on perceived stress in the community's older adults was insignificant.

## Data Availability

The data sets used and/or analyzed during the current study are available from the corresponding author on reasonable request.

## Abbreviations

ANOVA: Analysis of Variance
GEE: Generalized estimating equations
ADLs: Activities of Daily Living
TUG: Timed Up-and-Go Test
BBS: Berg Balance Scale
FR: Functional Reach Test
SPPB: Short Physical Performance Battery
BT: Balance Test
GS: Gait Speed
STS: Chair Sit-to-stand
OLST: One Leg Stand Test
AAQ: Attitudes to Aging Questionnaire
CPSS: Chinese Perceived Stress Scale
